# The Dice measure of cubic hesitant fuzzy sets and its initial evaluation method of benign prostatic hyperplasia symptoms

**DOI:** 10.1038/s41598-018-37228-9

**Published:** 2019-01-11

**Authors:** Jing Fu, Jun Ye, Wenhua Cui

**Affiliations:** 1 Shaoxing Second Hospital, 123 Yanan Road, Shaoxing, Zhejiang 312000 P. R. China; 2 Department of Electrical and Information Engineering, Shaoxing University, 508 Huancheng West Road, Shaoxing, Zhejiang 312000 P. R. China

## Abstract

In order to give the initial evaluation of benign prostatic hyperplasia (BPH) symptoms regarding a patient, physician usually performs the clinical inquiry of the patient, whereas his/her responses may contain hesitant fuzzy and uncertain information. However, existing evaluation/diagnosis approaches of BPH symptoms cannot cope with the hybrid problem of both hesitant and uncertain responses of patients. Furthermore, existing evaluation approaches may lose some useful responses (e.g. hesitant fuzzy information) so as to result in the unreasonable or indeterminate evaluation/diagnosis in the evaluation process of patients. To overcome this insufficiency, this study firstly introduces the concept of a cubic hesitant fuzzy set (CHFS) based on combining uncertain/interval-valued fuzzy information with hesitant fuzzy information so as to express the hybrid fuzzy information and proposes the Dice measure between CHFSs based on the extension method of the least common multiple cardinality/number (LCMC) for the hesitant fuzzy sets in CHFS. Then, the initial evaluation approach of BPH symptoms is developed by using the Dice measure of CHFSs. Lastly, the assessment results of six BPH patients are presented as the clinical actual cases to indicate the applicability and effectiveness of the developed assessment approach in CHFS setting. The comparison with existing common evaluation methods shows that the developed evaluation/diagnosis method is superior to the existing common evaluation methods in the evaluation/diagnosis process of the clinical actual cases.

## Introduction

In order to give the initial evaluation/diagnosis of a patient’s disease and symptoms, physician usually carries out the clinical inquiry of the patient, whereas the responses of the patient may contain fuzzy information due to his/her uncertainty and vagueness. Hence, the fuzzy medical diagnosis is an important research topic. It is a medical diagnosis method based on fuzzy relations of diseases and symptoms^1–3^. Due to the uncertainty of medical diagnosis information, a medical diagnosis approach was presented based on interval-valued fuzzy sets (IVFSs)^4^. To express the truth and falsity information, intuitionistic fuzzy sets and interval-valued intuitionistic fuzzy sets were applied to medical diagnoses^5–7^. To cope with medical diagnosis problems containing incomplete, uncertainty, and inconsistent information for a disease, simplified neutrosophic sets (SNSs), including single-value and interval neutrosophic sets, were applied to medical assessment/diagnosis problems^8–10^. Additionally, single valued neutrosophic multisets (refined neutrosophic sets) were applied in medical diagnosis problems^11–14^. Since there exist physicians’ hesitant thinking and representation in medical diagnosis problems, hesitant fuzzy sets (HFSs) were also applied to medical diagnosis problems^15,16^.

Since physicians’ thinking and expression may imply uncertain and hesitant evaluation information between a disease and symptoms in medical diagnosis process, however, the aforementioned diagnosis methods cannot cope with the evaluation/diagnosis problems with both uncertain information and hesitant information. Hence, they often lead to diagnostic confusion/uncertainty and puzzle due to losing some useful information. Furthermore, existing (fuzzy) cubic set (CS)^17^ can only represent the hybrid information of both an uncertain/interval-valued fuzzy number (IVFN) and a fuzzy value in real life, but not express the hybrid fuzzy information of both the uncertain/IVFN and the HFS composed of several possible fuzzy values. For instance, when three physicians are required to assess the severe degree of benign prostatic hyperplasia (BPH) symptoms for a patient, the IVFN [0.5, 0.7] is given by one of three experts and the HFS {0.5, 0.6} is given by two of three experts under their uncertain and hesitant situation, and then the hybrid form of both [0.5, 0.7] (the uncertain part) and {0.5, 0.6} (the hesitant fuzzy part) cannot be expressed simultaneously by the aforementioned various fuzzy concepts. Then, Fu *et al*.^18^ presented a cubic hesitant fuzzy set (CHFS) so as to express the hybrid fuzzy information and applied it to the evaluation/diagnosis problems of the prostate cancer in CHFS setting. However, the evaluation method in^18^ can only cope with the evaluation/diagnosis problems of the prostate cancer, but cannot suit the evaluation problems of BPH patients in CHFS setting. Furthermore, existing common evaluation methods of BPH symptoms^10,19,20^ can also cope with evaluation/diagnosis problems of BPH symptoms with SNSs or uncertain information, but cannot handle evaluation/diagnosis problems of BPH symptoms with both uncertain and hesitant fuzzy information in CHFS setting. As the further generalization of the cubic hesitant fuzzy evaluation/diagnosis method^18^, this study extends it to the evaluation problems of BPH patients. To do so, this paper first proposes the Dice measure of CHFSs, and then develops the initial evaluation/diagnosis of BPH symptoms based on the Dice measure of CHFSs to solve the initial evaluation/diagnosis problems of BPH symptoms in CHFS setting.

As the framework of this study, Section 2 introduces the CHFS concept based on the hybrid form of both IVFN and HFS and proposes the Dice measure between CHFSs based on the extension method of the least common multiple cardinality/number (LCMC) for the HFSs in CHFS. In Section 3, the evaluation method of BPH symptoms is developed based on the Dice measure of CHFSs under CHFS setting. Section 4 presents the evaluation of the BPH symptoms by six BPH patients as the clinical actual cases to show the effectiveness and rationality of the evaluation approach of BPH symptoms based on the Dice measure of CHFSs. Lastly, conclusions and further study are contained in Section 5.

## Cubic hesitant fuzzy sets and the Dice measure of CHFSs

### Cubic hesitant fuzzy sets

Regarding a hybrid form of an IVFN and a fuzzy value, Jun *et al*.^17^ presented a (fuzzy) CS in a fixed non-empty set *U* by the following form:

$$C=\{\langle u,\tilde{a}(u),\mu (u)\rangle |u\in U\},$$

where *a*(*u*) = [*a*^*−*^, *a*^+^] is an IVFN and *μ*(*u*) is a fuzzy value for *u* ∈ *U*.

Then, a HFS concept^21,22^ in a fixed non-empty set *U* is expressed as

$$B=\{u,\,\tilde{h}(u)|u\in U\},$$

where $\tilde{h}(u)$ is a set of several different values in [0, 1], denoted by $\tilde{h}(u)$ = {*μ*_1_, *μ*_2_, …, *μ*_*t*_} for *u* ∈ *U*.

Regarding a hybrid form of both a HFS and a CS, Fu *et al*.^18^ gave the definition of CHFS below.

#### **Definition 1**

. Set *U* as a fixed non-empty set. A CHFS *R* is defined as the following form^18^:

$$R=\{\langle u,\,{\tilde{a}}_{R}(u),\,{\tilde{h}}_{R}(u)\rangle |u\in U\},$$

where ${\tilde{a}}_{R}(u)$ for *u* ∈ *U* is an IVFN for ${\tilde{a}}_{R}(u)=[{a}^{-},\,{a}^{+}]$ ⊆ [0, 1], and ${\tilde{h}}_{R}(u)$ for *u* ∈ *U* is a HFS, which contains several different fuzzy values in [0, 1] expressed by ${\tilde{h}}_{R}(u)$ = {*μ*_1_, *μ*_2_, …, *μ*_*t*_} in an ascending order.

Then, the basic element $\langle u,{\tilde{a}}_{R}(u),{\tilde{h}}_{R}(u)\rangle$ in *R* is denoted simply as $r=\langle \tilde{a},\,\tilde{h}\rangle =\langle [{a}^{-},\,{a}^{+}],\{{\mu }_{1},\,{\mu }_{2},\,\mathrm{...},\,{\mu }_{t}\}\rangle$ for expressional convenience, which is called as a cubic hesitant fuzzy element (CHFE).

Especially when *t* = 1, CHFS is reduced to CS, which is a special case of CHFS.

#### **Definition 2**

. Set $r=\langle [{a}^{-},\,{a}^{+}],\,\{{\mu }_{1},\,{\mu }_{2},\,\mathrm{...},\,{\mu }_{t}\}\rangle$ as a CHFE, then one call it^18^

- An internal CHFE if every *μ*_*k*_ ∈ [*a*^−^, *a*^+^] for *k* = 1, 2, …, *t*;
- An external CHFE if every *μ*_*k*_ ∉ [*a*^−^, *a*^+^] for *k* = 1, 2, …, *t*.

For example, *r* = 〈[0.5, 0.7], {0.6, 0.7}〉 is called an internal CHFE, where [0.5, 0.7] is its IVFN and {0.6, 0.7} is its HFS.

#### **Definition 3**

. Set ${r}_{1}=\langle {\tilde{a}}_{1},\,{\tilde{h}}_{1}\rangle =\langle [{a}_{1}^{-},\,{a}_{1}^{+}],\{{\mu }_{11},\,{\mu }_{12},\,\mathrm{...},\,{\mu }_{1t}\}\rangle$ and ${r}_{2}=\langle {\tilde{a}}_{2},\,{\tilde{h}}_{2}\rangle =\langle [{a}_{2}^{-},{a}_{2}^{+}],\{{\mu }_{21},\,{\mu }_{22},\,\mathrm{...},$ ${\mu }_{2t}\}\rangle$ as two CHFEs, then there exist the following relations^18^:

(i) *r*_1_ = r_2_ ⇔ ${\tilde{a}}_{1}={\tilde{a}}_{2}$ and ${\tilde{h}}_{1}={\tilde{h}}_{2}$, i.e., ${a}_{1}^{-}={a}_{2}^{-}$, ${a}_{1}^{+}={a}_{2}^{+}$, and ${\mu }_{1k}={\mu }_{2k}$ for *k* = 1, 2, …, *t*;
(ii) *r*_1_ ⊆ *r*_2_ ⇔ ${\tilde{a}}_{1}\subseteq {\tilde{a}}_{2}$ and ${\tilde{h}}_{1}\subseteq {\tilde{h}}_{2}$, i.e., *μ*_1*k*_ ≤ *μ*_2*k*_ for *k* = 1, 2, …, *t*;
(iii) ${r}_{1}^{c}=\langle {\tilde{a}}_{1}^{c},{\tilde{h}}_{1}^{c}\rangle =\langle [1-{a}_{1}^{+},1-{a}_{1}^{-}],\{1-{\mu }_{1t},1-{\mu }_{1t-1},\mathrm{...},1-{\mu }_{11}\}\rangle$ as the complement of *r*_1_.

Generally speaking, for two different CHFEs ${\tilde{h}}_{1}$ and ${\tilde{h}}_{2}$ the cardinality (the number of components) between two HFSs ${\tilde{h}}_{1}$ and ${\tilde{h}}_{2}$ may imply difference. Thus, the two HFSs ${\tilde{h}}_{1}$ and ${\tilde{h}}_{2}$ are extended based on the LCMC extension method^18^ until both reach the same cardinality (the same number of components) so as to reach reasonable operations of two different CHFEs. Obviously, this LCMC extension method shows the advantage of objectivity and feasibility.

Assume two CHFEs are ${r}_{1}=\langle {\tilde{a}}_{1},{\tilde{h}}_{1}\rangle =\langle [{a}_{1}^{-},{a}_{1}^{+}],\{{\mu }_{11},{\mu }_{12},\mathrm{...},{\mu }_{1{t}_{1}}\}\rangle$ and ${r}_{2}=\langle {\tilde{a}}_{2},{\tilde{h}}_{2}\rangle =\langle [{a}_{2}^{-},{a}_{2}^{+}],\{{\mu }_{21},$ ${\mu }_{22},\mathrm{...},{\mu }_{2{t}_{2}}\}\rangle$ and the LCMC of *t*_1_ and *t*_2_ in ${\tilde{h}}_{1}$ and ${\tilde{h}}_{2}$ is *q*. Then both can be extended into the following forms:

1 $${r}_{1}^{e}=\langle [{a}_{1}^{-},{a}_{1}^{+}],\{\mathop{\overbrace{{\lambda }_{11}^{1},{\lambda }_{11}^{2},\ldots ,{\lambda }_{11}^{q/{t}_{1}},{\lambda }_{12}^{1},{\lambda }_{12}^{2},\ldots ,{\lambda }_{12}^{q/{t}_{1}},\mathrm{....},{\lambda }_{1{t}_{1}}^{1},{\lambda }_{1{t}_{1}}^{2},\ldots ,{\lambda }_{1{t}_{1}}^{q/{t}_{1}}}}\limits^{q}\}\rangle ,$$

2 $${r}_{2}^{e}=\langle [{a}_{2}^{-},{a}_{2}^{+}],\{\mathop{\overbrace{{\lambda }_{21}^{1},{\lambda }_{21}^{2},\ldots ,{\lambda }_{21}^{q/{t}_{2}},{\lambda }_{22}^{1},{\lambda }_{22}^{2},\ldots ,{\lambda }_{22}^{q/{t}_{2}},\mathrm{....},{\lambda }_{2{t}_{2}}^{1},{\lambda }_{2{t}_{2}}^{2},\ldots ,{\lambda }_{2{t}_{2}}^{q/{t}_{2}}}}\limits^{q}\}\rangle .$$

For convenient representation, Eqs (1) and (2) are also written as the following simple form:

3 $${r}_{1}^{e}=\langle [{a}_{1}^{-},{a}_{1}^{+}],\{{\lambda }_{1}^{(1)},{\lambda }_{1}^{(2)},\ldots ,{\lambda }_{1}^{(q)}\}\rangle ,$$

4 $${r}_{2}^{e}=\langle [{a}_{2}^{-},{a}_{2}^{+}],\{{\lambda }_{2}^{(1)},{\lambda }_{2}^{(2)},\ldots ,{\lambda }_{2}^{(q)}\}\rangle .$$

The following numerical example is given to indicate the LCMC extension method.

#### **Example 1**.

Let *r*_1_ = <[0.5, 0.8], {0.6, 0.7}> and *r*_2_ = <[0.3, 0.6], {0.3, 0.4, 0.5}> be two CHFEs. They are extended by the LCMC extension method.

The LCMC of both is *q* = 6 for *t*_1_ = 2 and *t*_2_ = 3 in *r*_1_ and *r*_2_. By applying Eqs (1) and (2), the two CHFEs *h*_1_ and *h*_2_ can be extended to the following forms:

$${r}_{1}^{e}=\langle [0.5,0.8],\{0.6,0.6,0.6,0.7,0.7,0.7\}\rangle \,{\rm{and}}\,{r}_{2}^{e}=\langle [0.3,0.6],\{0.3,0.3,0.4,0.4,0.5,0.5\}\rangle .$$

### The Dice measure of CHFSs

In this subsection, we propose the Dice measure of CHFSs based on the LCMC extension method for the HFSs in CHFS since the similarity measure is an important mathematical tool in pattern recognition and medical diagnosis areas.

#### **Definition 4**.

Set *R*_1_ = {*r*_11_, *r*_12_, …, *r*_1*n*_} and *R*_2_ = {*r*_21_, *r*_22_, …, *r*_2*n*_} as two CHFSs, where ${r}_{1k}=\langle {\tilde{a}}_{1k},{\tilde{h}}_{1k}\rangle =\langle [{a}_{1k}^{-},{a}_{1k}^{+}],$ $\{{\mu }_{1k}^{(1)},{\mu }_{1k}^{(2)},\mathrm{...},{\mu }_{1k}^{({q}_{k})}\}\rangle$ and ${r}_{2k}=\langle {\tilde{a}}_{2k},{\tilde{h}}_{2k}\rangle =\langle [{a}_{2k}^{-},{a}_{2k}^{+}],\{{\mu }_{2k}^{(1)},{\mu }_{2k}^{(2)},\mathrm{...},{\mu }_{2k}^{({q}_{k})}\}\rangle$ with their LCMC *q*_*k*_ (*k* = 1, 2, …, *n*) are CHFEs. If *r*_1*n*_ and *r*_2*n*_ are considered as the two vectors of *q*_*k*_ + 2 dimensions, the Dice measure between *R*_1_ and *R*_2_ is defined as

5 $$\begin{array}{ccc}D({R}_{1},{R}_{2}) & = & \frac{1}{n}\sum _{k=1}^{n}\frac{2{r}_{1k}\cdot {r}_{2k}}{{\Vert {r}_{1k}\Vert }^{2}+{\Vert {r}_{2k}\Vert }^{2}}\\  & = & \frac{1}{n}\sum _{k=1}^{n}\tfrac{2({a}_{1k}^{-}{a}_{2k}^{-}+{a}_{1k}^{+}{a}_{2k}^{+}+{\mu }_{1k}^{(1)}{\mu }_{2k}^{(1)}+{\mu }_{1k}^{(2)}{\mu }_{2k}^{(2)}+\mathrm{...}+{\mu }_{1k}^{({q}_{k})}{\mu }_{2k}^{({q}_{k})})}{[{({a}_{1k}^{-})}^{2}+{({a}_{1k}^{+})}^{2}+{({\mu }_{1k}^{(1)})}^{2}+{({\mu }_{1k}^{(2)})}^{2}+\mathrm{...}+{({\mu }_{1k}^{({q}_{k})})}^{2}]+[{({a}_{2k}^{-})}^{2}+{({a}_{2k}^{+})}^{2}+{({\mu }_{2k}^{(1)})}^{2}+{({\mu }_{2k}^{(2)})}^{2}+\mathrm{...}+{({\mu }_{2k}^{({q}_{k})})}^{2}]}\end{array}$$

Then, the Dice measure *D*(*R*_1_, *R*_2_) indicates the following proposition.

#### **Proposition 1**.

The Dice measure *D*(*R*_1_, *R*_2_) contains the following properties:

- 0 ≤ *D*(*R*_1_, *R*_2_) ≤ 1;
- *D*(*R*_1_, *R*_2_) = 1 ⇔ *R*_1_ = *R*_2_;
- *D*(*R*_1_, *R*_2_) = *D*(*R*_2_, *R*_1_).

**Proof:**

- Corresponding to the inequalities (*a* − *b*)^2^ ≥ 0 and *a*^2^ + *b*^2^ ≥ 2*ab*, the property (a) is true.
- If *r*_1*k*_ = *r*_2*k*_, then there are ${\tilde{a}}_{1k}={\tilde{a}}_{2k}$ and ${\tilde{h}}_{1k}={\tilde{h}}_{2k}$, i.e., ${a}_{1k}^{-}={a}_{2k}^{-}$, ${a}_{1k}^{+}={a}_{2k}^{+}$, and ${\mu }_{1k}^{({q}_{k})}={\mu }_{2k}^{({q}_{k})}$ for *k* = 1, 2, …, *n*. Hence, *D*(*R*_1_, *R*_2_) = 1. On the contrary, if *D*(*R*_1_, *R*_2_) = 1, then there are *r*_1*k*_ = *r*_2*k*_, i.e., ${\tilde{a}}_{1k}={\tilde{a}}_{2k}$ and ${\tilde{h}}_{1k}={\tilde{h}}_{2k}$. Thus there are ${a}_{1k}^{-}={a}_{2k}^{-}$, ${a}_{1k}^{+}={a}_{2k}^{+}$, and ${\mu }_{1k}^{\,({q}_{k})}={\mu }_{2k}^{({q}_{k})}$ for *k* = 1, 2, …, *n*. Hence, *R*_1_ = *R*_2_ can hold.
- It is obvious that the property (c) is true.

When the importance of the CHFEs *r*_1*k*_ and *r*_2*k*_ is taken into account, we set *ω*_*k*_ for 0 ≤ *ω*_*k*_ ≤ 1 and ${\sum }_{k=1}^{n}{\omega }_{k}=1$ as the weight of the CHFEs *r*_1*k*_ and *r*_2*k*_. Thus, the weighted Dice measure between *R*_1_ and *R*_2_ is presented as

6 $$\begin{array}{ccc}{D}_{\omega }({R}_{1},{R}_{2}) & = & \sum _{k=1}^{n}{\omega }_{k}\frac{2{r}_{1k}\cdot {r}_{2k}}{{\Vert {r}_{1k}\Vert }^{2}+{\Vert {r}_{2k}\Vert }^{2}}\\  & = & \sum _{k=1}^{n}{\omega }_{k}\tfrac{2({a}_{1k}^{-}{a}_{2k}^{-}+{a}_{1k}^{+}{a}_{2k}^{+}+{\mu }_{1k}^{(1)}{\mu }_{2k}^{(1)}+{\mu }_{1k}^{(2)}{\mu }_{2k}^{(2)}+\mathrm{...}+{\mu }_{1k}^{({q}_{k})}{\mu }_{2k}^{({q}_{k})})}{[{({a}_{1k}^{-})}^{2}+{({a}_{1k}^{+})}^{2}+{({\mu }_{1k}^{(1)})}^{2}+{({\mu }_{1k}^{(2)})}^{2}+\mathrm{...}+{({\mu }_{1k}^{({q}_{k})})}^{2}]+[{({a}_{2k}^{-})}^{2}+{({a}_{2k}^{+})}^{2}+{({\mu }_{2k}^{(1)})}^{2}+{({\mu }_{2k}^{(2)})}^{2}+\mathrm{...}+{({\mu }_{2k}^{({q}_{k})})}^{2}]}\end{array}$$

Thus, the weighted Dice measure *D*_*ω*_(*R*_1_, *R*_2_) also has the following proposition.

#### **Proposition 2**.

The weighted Dice measure *D*_*ω*_(*R*_1_, *R*_2_) contains the following properties:

- 0 ≤ *D*_*ω*_(*R*_1_, *R*_2_) ≤ 1;
- *D*_*ω*_(*R*_1_, *R*_2_) = 1 ⇔ *R*_1_ = *R*_2_;
- *D*_*ω*_(*R*_1_, *R*_2_) = *D*_*ω*_(*R*_2_, *R*_1_).

By the similar proof manner of Proposition 1, we can prove Proposition 2, which is omitted here.

#### **Example 2**.

Let us consider two CHFSs:

*R*_1_ = {*r*_11_, *r*_12_} = {<[0.6, 0.7], {0.5, 0.6}>, <[0.3, 0.5], {0.3, 0.4, 0.5}>},

*R*_2_ = {*r*_21_, *r*_22_} = {<[0.3, 0.6], {0.4, 0.5, 0.6}>, <[0.6, 0.8], {0.7, 0.8}>}.

Then, their weight vector is given as *ω* = (0.4, 0.6) to calculate the weighted Dice measure between *R*_1_ and *R*_2_.

First, we get their LCMC *q*_1_ = *q*_2_ = 6 from a pair of *r*_11_ and *r*_21_ and a pair of *r*_12_ and *r*_22_. Thus, we get the following extension forms:

*R*_1_ = $$\{{r}_{11}^{e},{r}_{12}^{e}\}$$ = {<[0.6, 0.7], {0.5, 0.5, 0.5, 0.6, 0.6, 0.6}>, <[0.3, 0.5], {0.3, 0.3, 0.4, 0.4, 0.5, 0.5}>},

*R*_2_ = $$\{{r}_{21}^{e},{r}_{22}^{e}\}$$ = {<[0.3, 0.6], {0.4, 0.4, 0.5, 0.5, 0.6, 0.6}>, <[0.6, 0.8], {0.7, 0.7, 0.7, 0.8, 0.8, 0.8}>}.

Then, the weighted Dice measure between *R*_1_ and *R*_2_ is calculated by the following form:

$$\begin{array}{l}{D}_{\omega }({R}_{1},{R}_{2})\\ \begin{array}{rcl} & = & \sum _{k=1}^{2}\,{\omega }_{k}\tfrac{2({a}_{1k}^{-}{a}_{2k}^{-}+{a}_{1k}^{+}{a}_{2k}^{+}+{\mu }_{1k}^{(1)}{\mu }_{2k}^{(1)}+{\mu }_{1k}^{(2)}{\mu }_{2k}^{(2)}+\cdots +{\mu }_{1k}^{({q}_{k})}{\mu }_{2k}^{({q}_{k})})}{[{({a}_{1k}^{-})}^{2}+{({a}_{1k}^{+})}^{2}+{({\mu }_{1k}^{(1)})}^{2}+{({\mu }_{1k}^{(2)})}^{2}+\cdots +{({\mu }_{1k}^{({q}_{k})})}^{2}]+[{({a}_{2k}^{-})}^{2}+{({a}_{2k}^{+})}^{2}+{({\mu }_{2k}^{(1)})}^{2}+{({\mu }_{2k}^{(2)})}^{2}+\cdots +{({\mu }_{2k}^{({q}_{k})})}^{2}]}\\  & = & \tfrac{0.4\times 2\times (0.6\times 0.3+0.7\times 0.6+0.5\times 0.4+0.5\times 0.4+0.5\times 0.5+0.6\times 0.5+0.6\times 0.6+0.6\times 0.6}{({0.6}^{2}+{0.7}^{2}+{0.5}^{2}+{0.5}^{2}+{0.5}^{2}+{0.6}^{2}+{0.6}^{2}+{0.6}^{2})+({0.3}^{2}+{0.6}^{2}+{0.4}^{2}+{0.4}^{2}+{0.5}^{2}+{0.5}^{2}+{0.6}^{2}+{0.6}^{2})}\\  &  & +\,\tfrac{0.6\times 2\times (0.3\times 0.6+0.5\times 0.8+0.3\times 0.7+0.3\times 0.7+0.4\times 0.7+0.4\times 0.8+0.5\times 0.8+0.5\times 0.8}{({0.3}^{2}+{0.5}^{2}+{0.3}^{2}+{0.3}^{2}+{0.4}^{2}+{0.4}^{2}+{0.5}^{2}+{0.5}^{2})+({0.6}^{2}+{0.8}^{2}+{0.7}^{2}+{0.7}^{2}+{0.7}^{2}+{0.8}^{2}+{0.8}^{2}+{0.8}^{2})}\\  & = & \mathrm{0.8915.}\end{array}\end{array}$$

## The Dice measure-based evaluation/diagnosis method of BPH symptoms

Aging men commonly encounter the disease of BPH and suffer from obstructive and irritative voiding symptoms. To assess BPH symptoms, the seven questions introduced by the American Urological Association (AUA) are considered as the AUA symptom indices^19,20^ for BPH, which are scored on a scale from 0 to 5 points so as to use the evaluation/diagnosis of BPH symptoms for clinical patients. An objective documentation of BPH symptoms was offered by the international prostate symptom score (I-PSS)^19,20^, In existing common clinical evaluation/diagnosis of BPH symptoms, the common score and evaluation method^19,20^ are shown in Tables 1 and 2. Then, the total score in Table 1 was thirty-five, which can be classified into three types of evaluation grades of BPH symptoms in Table 2, along with totally scoring 0–7 as mild symptom, 8–19 as moderate symptom, and 20–35 as severe symptom for a BPH patient.

**Table 1:** The score of BPH symptoms in 5 times over the past month for BPH patients based on I-PSS^19,20^.

*A*_*i*_	Score of one time	Score of two times	Score of three times	Score of four times	Score of five times
*A*_1_ *(How often have you had a sensation of not emptying your bladder completely after you finished urinating?)*	1	2	3	4	5
*A*_2_ *(How often have you had to urinating again less than two hours after you finished urinating?)*	1	2	3	4	5
*A*_3_ *(How often have you found you stopped and started again several times when you urinated?)*	1	2	3	4	5
*A*_4_ *(How often have you found it difficult to postpone urination?)*	1	2	3	4	5
*A*_5_ *(How often have you had a week urinary stream?)*	1	2	3	4	5
*A*_6_ *(How often have you had to push or strain to begin urination?)*	1	2	3	4	5
*A*_7_ *(How many times did you most typically get up to urinate from the time you went to bed at night until the time you got up in the morning?)*	1	2	3	4	5

**Table 2:** The common evaluation/diagnosis classification given based on I-PSS^19,20^.

	*R*_1_	*R*_2_	*R*_3_
Totally scoring value	0–7	8–19	20–35
BPH symptom	Mild symptom	Moderate symptom	Severe symptom

However, this objective evaluation method presented in I-PSS is a traditional and non-fuzzy assessment/diagnosis, whereas the patient’s response to the seven questions of BPH symptoms may imply the hybrid information of both uncertain responses and hesitant responses regarding his/her vague symptoms indicated over the past month. Obviously, CHFS is very fit for the expression of the hybrid information. Thus, the Dice measure-based evaluation/diagnosis can solve the evaluation/diagnosis problems of BPH symptoms with CHFS information. Therefore, this section proposes the Dice measure-based evaluation/diagnosis approach of BPH symptoms in CHFS setting.

Based on Table 1, this study firstly establishes the inquiry table of BPH symptoms with uncertain and hesitant arguments, as shown in Table 3. In Table 3, a collection of the seven questions is expressed by the set of attributes/indices *A* = {*A*_1_, *A*_2_, *A*_3_, *A*_4_, *A*_5_, *A*_6_, *A*_7_} and the clinical inquiry and answer of a patient *Q*_*i*_ (*i* = 1, 2, …, *t*) indicate the BPH symptom responses in 5 times over the past month. However, since the patient’s answers may imply his/her uncertainty and hesitancy corresponding to the seven questions, he/she can give the uncertain range and hesitant values in his/her BPH symptom responses in 5 times over the past month.

**Table 3:** BPH symptom responses in 5 times for a patient *Q*_*i*_ over the past month.

Question	Answer	Answer
*A*_1_ (How often have you had a sensation of not emptying your bladder completely after you finished urinating?)	Uncertain range	Hesitant value
*A*_2_ (How often have you had to urinating again less than two hours after you finished urinating?)	Uncertain range	Hesitant value
*A*_3_ (How often have you found you stopped and started again several times when you urinated?)	Uncertain range	Hesitant value
*A*_4_ (How often have you found it difficult to postpone urination?)	Uncertain range	Hesitant value
*A*_5_ (How often have you had a week urinary stream?)	Uncertain range	Hesitant value
*A*_6_ (How often have you had to push or strain to begin urination?)	Uncertain range	Hesitant value
*A*_7_ (How many times did you most typically get up to urinate from the time you went to bed at night until the time you got up in the morning?)	Uncertain range	Hesitant value

In the clinical actual application, we require that physicians ask the BPH symptoms of patients over the past month by the seven questions in Table 3 so as to obtain the uncertain and hesitant information from a BPH patient *Q*_*i*_.

Regarding I-PSS^19,20^, we can also sort BPH patients into the three types of symptoms: Mild symptom (*R*_1_), Moderate symptom (*R*_2_), and Severe symptom (*R*_3_), which are constructed as a set of the three types of symptoms *R* = {*R*_1_, *R*_2_, *R*_3_}, indicating the three symptom patterns, to be used for the initial evaluation/diagnosis of BPH patients, as shown in Table 4.

**Table 4:** Three patterns of the BPH symptoms with CHFEs.

*R*_*i*_	*A*_1_	*A*_2_	*A*_3_	*A*_4_	*A*_5_	*A*_6_	*A*_7_
*R*_1_ (Mild symptom)	<[0, 0.2], {0, 0.2}>	<[0, 0.2], {0, 0.2}>	<[0, 0.2], {0, 0.2}>	<[0, 0.2], {0, 0.2}>	<[0, 0.2], {0, 0.2}>	<[0, 0.2], {0, 0.2}>	<[0, 0.2], {0, 0.2}>
*R*_2_ (Moderate symptom)	<[0.2, 0.5], {0.3, 0.4}>	<[0.2, 0.5], {0.3, 0.4}>	<[0.2, 0.5], {0.3, 0.4}>	<[0.2, 0.5], {0.3, 0.4}>	<[0.2, 0.5], {0.3, 0.4}>	<[0.2, 0.5], {0.3, 0.4}>	<[0.2, 0.5], {0.3, 0.4}>
*R*_3_ (Severe symptom)	<[0.6, 1], {0.75, 0.85}>	<[0.6, 1], {0.75, 0.85}>	<[0.6, 1], {0.75, 0.85}>	<[0.6, 1], {0.75, 0.85}>	<[0.6, 1], {0.75, 0.85}>	<[0.6, 1], {0.75, 0.85}>	<[0.6, 1], {0.75, 0.85}>

In Table 4, the three symptom patterns of BPH patients regarding the seven questions can be expressed as the following CHFSs:

*R*_1_ = {〈*A*_1_, [0, 0.2], {0, 0.2}〉, 〈*A*_2_, [0, 0.2], {0, 0.2}〉, 〈*A*_3_, [0, 0.2], {0, 0.2}〉, 〈*A*_4_, [0, 0.2], {0, 0.2}〉, 〈*A*_5_, [0, 0.2], {0, 0.2}〉, 〈*A*_6_, [0, 0.2], {0, 0.2}〉, 〈*A*_7_, [0, 0.2], {0, 0.2}〉},

*R*_2_ = {〈*A*_1_, [0.2, 0.5], {0.3,0.4}〉, 〈*A*_2_, [0.2, 0.5], {0.3, 0.4}〉, 〈*A*_3_, [0.2, 0.5], {0.3, 0.4}〉, 〈*A*_4_, [0.2, 0.5], {0.3, 0.4}〉, 〈*A*_5_, [0.2, 0.5], {0.3, 0.4}〉, 〈*A*_6_, [0.2, 0.5], {0.3, 0.4}〉, 〈*A*_7_, [0.2, 0.5], {0.3, 0.4}〉},

*R*_3_ = {〈*A*_1_, [0.6, 1], {0.75, 0.85}〉, 〈*A*_2_, [0.6, 1], {0.75, 0.85}〉, 〈*A*_3_, [0.6, 1], {0.75, 0.85}〉, 〈*A*_4_, [0.6, 1], {0.75, 0.85}〉, 〈*A*_5_, [0.6, 1], {0.75, 0.85}〉, 〈*A*_6_, [0.6, 1], {0.75, 0.85}〉, 〈*A*_7_, [0.6, 1], {0.75, 0.85}〉}.

Suppose that the clinical inquiries and answers of *t* BPH patients are obtained by Table 3, then we can transform both uncertain ranges and hesitant values into the form of CHFEs. For a patient *Q*_*i*_ (*i* = 1, 2, …, *t*) corresponding to CHFE information, we can present the following evaluation approach.

The Dice measure *D*_*ω*_(*Q*_*i*_, *R*_*j*_) for *j* = 1, 2, 3 and *i* = 1, 2, …, *t* can be calculated in order to obtain a fit evaluation/diagnosis of a BPH patient *Q*_*i*_. Then, the fit evaluation *R*_j*_ of the BPH patient *Q*_*i*_ can be yielded by ${j}^{\ast }={\rm{\arg }}\mathop{{\rm{\max }}}\limits_{1\le j\le 3}$ $\{{D}_{\omega }({Q}_{i},{R}_{j})\}$.

## Actual evaluation cases of BPH symptoms

In this section, we consider the clinical actual cases regarding six BPH patients to show the evaluation process of BPH symptoms by the evaluation/diagnosis method of BPH symptoms based on the Dice measure of CHFSs.

First, the six BPH patients *Q*_*i*_ (*i* = 1, 2, …, 6) in the clinical actual cases indicate their responses to the clinical inquiries from Table 3, which are shown in Table 5.

**Table 5:** BPH symptom responses of the six clinical patients in 5 times over the past month.

*A*_*i*_	*Q*_1_		*Q*_2_		*Q*_3_		*Q*_4_		*Q*_5_		*Q*_6_	
	Uncertain value	Hesitant value	Uncertain value	Hesitant value	Uncertain value	Hesitant value	Uncertain value	Hesitant value	Uncertain value	Hesitant value	Uncertain value	Hesitant value
*A*_1_	2–4	3	0-1	0, 1	1–3	2	2–4	3	3-4	3, 4	2-3	2, 3
*A*_2_	3–5	4	0-1	0, 1	0-1	0, 1	2–4	3	3-4	3, 4	2-3	2, 3
*A*_3_	2-3	2, 3	1	1	0–2	1	1–3	2	3–5	4	1–2	1, 2
*A*_4_	2–4	3	0–1	0, 1	1–2	1–2	3	3	3–5	4	2–3	2, 3
*A*_5_	3–5	4	0–1	0, 1	2–4	3	3–4	3, 4	3–5	4	1–2	1, 2
*A*_6_	2–3	2, 3	1	1	2–3	2–3	3	3	3–5	4	2–3	2, 3
*A*_7_	2–4	3	0–1	0, 1	1–4	2–3	3–4	3, 4	2–3	2, 3	2–3	2, 3

Based on the inquiry results in Table 5, the normalized response values are obtained corresponding to the response times (uncertain values and hesitant values) divided by 5, and then can be transformed into CHFEs, which are shown in Table 6.

**Table 6:** All the CHFEs for the six BPH patients *Q*_*i*_ (*i* = 1, 2, …, 6).

*A*_*i*_	*Q*_1_	*Q*_2_	*Q*_3_	*Q*_4_	*Q*_5_	*Q*_6_
*A*_1_	([0.4, 0.8], {0.6})	([0, 0.2], {0, 0.2})	([0.2, 0.6], {0.4})	([0.4, 0.8], {0.6})	([0.6, 0.8], {0.6, 0.8})	([0.4, 0.6], {0.3, 0.6})
*A*_2_	([0.6, 1], {0.8})	([0, 0.2], {0, 0.2})	([0, 0.2], {0, 0.2})	([0.4, 0.8], {0.6})	([0.6, 0.8], {0.6, 0.8})	([0.4, 0.6], {0.4, 0.6})
*A*_3_	([0.4, 0.6], {0.4, 0.6})	([0.2, 0.2], {0.2})	([0, 0.4], {0.2})	([0.2, 0.6], {0.4})	([0.6, 1], {0.8})	([0.2, 0.4], {0.2, 0.4})
*A*_4_	([0.4, 0.8], {0.6})	([0, 0.2], {0, 0.2})	([0.2, 0.4], {0.2, 0.4})	([0.6, 0.6], {0.6})	([0.6, 1], {0.8})	([0.4, 0.6], {0.4, 0.6})
*A*_5_	([0.6, 1], {0.8})	([0, 0.2], {0, 0.2})	([0.4, 0.8], {0.6})	([0.6, 0.8], {0.6, 0.8})	([0.6, 1], {0.8})	([0.2, 0.4], {0.2, 0.4})
*A*_6_	([0.4, 0.6], {0.4, 0.6})	([0.2, 0.2], {0.2})	([0.4, 0.6], {0.4, 0.6})	([0.6, 0.6], {0.6})	([0.6, 1], {0.8})	([0.4, 0.6], {0.4, 0.6})
*A*_7_	([0.4, 0.8], {0.6})	([0, 0.2], {0, 0.2})	([0.2, 0.8], {0.4, 0.6})	([0.6, 0.8], {0.6, 0.8})	([0.4, 0.6], {0.4, 0.6})	([0.4, 0.6], {0.4, 0.6})

From Table 6, all the CHFEs regarding the BPH patients *Q*_*i*_ (*i* = 1, 2, …, 6) can be expressed as the extension CHFSs based on the LCMC of HFSs *q*_*k*_ = 2 (*k* = 1, 2, …, 7):

*Q*_1_ = {<*A*_1_, [0.4, 0.8], {0.6, 0.6}>, <*A*_2_, [0.6, 1], {0.8, 0.8}>, <*A*_3_, [0.4, 0.6], {0.4, 0.6}>, <*A*_4_, [0.4, 0.8], {0.6, 0.6}>, <*A*_5_, [0.6, 1], {0.8, 0.8}>, <*A*_6_, [0.4, 0.6], {0.4, 0.6}>, <*A*_7_, [0.4, 0.8], {0.6, 0.6}>},

*Q*_2_ = {<*A*_1_, [0, 0.2], {0, 0.2}>, <*A*_2_, [0, 0.2], {0, 0.2}>, <*A*_3_, [0.2, 0.2], {0.2, 0.2}>, <*A*_4_, [0, 0.2], {0, 0.2}>, <*A*_5_, [0, 0.2], {0, 0.2}>, <*A*_6_, [0.2, 0.2], {0.2, 0.2}>, <*A*_7_, [0, 0.2], {0, 0.2}>},

*Q*_3_ = {<*A*_1_, [0.2, 0.6], {0.4, 0.4}>, <*A*_2_, [0, 0.2], {0, 0.2}>, <*A*_3_, [0, 0.4], {0.2, 0.2}>, <*A*_4_, [0.2, 0.4], {0.2, 0.4}>, <*A*_5_, [0.4, 0.8], {0.6, 0.6}>, <*A*_6_, [0.4, 0.6], {0.4,0.6}>, <*A*_7_, [0.2, 0.8], {0.4, 0.6}>},

*Q*_4_ = {<*A*_1_, [0.4, 0.8], {0.6, 0.6}>, <*A*_2_, [0.4, 0.8], {0.6, 0.6}>, <*A*_3_, [0.2, 0.6], {0.4, 0.4}>, <*A*_4_, [0.6, 0.6], {0.6, 0.6}>, <*A*_5_, [0.6, 0.8], {0.6, 0.8}>, <*A*_6_, [0.6, 0.6], {0.6, 0.6}>, <*A*_7_, [0.6, 0.8], {0.6, 0.8}>},

*Q*_5_ = {<*A*_1_, [0.6, 0.8], {0.6, 0.8}>, <*A*_2_, [0.6, 0.8], {0.6, 0.8}>, <*A*_3_, [0.6, 1], {0.8, 0.8}>, <*A*_4_, [0.6, 1], {0.8, 0.8}>, <*A*_5_, [0.6, 1], {0.8, 0.8}>, <*A*_6_, [0.6, 1], {0.8, 0.8}>, <*A*_7_, [0.4, 0.6], {0.4, 0.6}>},

*Q*_6_ = {<*A*_1_, [0.4, 0.6], {0.4, 0.6}>, <*A*_2_, [0.4, 0.6], {0.4, 0.6}>, <*A*_3_, [0.2, 0.4], {0.2, 0.4}>, <*A*_4_, [0.4, 0.6], {0.4, 0.6}>, <*A*_5_, [0.2, 0.4], {0.2, 0.4}>, <*A*_6_, [0.4, 0.6], {0.4, 0.6}>, <*A*_7_, [0.4, 0.6], {0.4, 0.6}>}.

Suppose the weight of each element *A*_*k*_ is *ω*_*k*_ = 1/7 for *k* = 1, 2, …, 7. By using Eq. (6), we can yield the Dice measure results between the patient *Q*_*i*_ (*i* = 1, 2, …, 6) and the symptom pattern *R*_*j*_ (*j* = 1, 2, 3), which are shown in Table 7.

**Table 7:** The Dice measure values between *Q*_*i*_ and *R*_*j*_ with CHFSs.

	*R*_1_	*R*_2_	*R*_3_
*D*_*ω*_(*Q*_1_, *R*_*j*_)	0.3338	0.8542	**0.9529**
*D*_*ω*_(*Q*_2_, *R*_*j*_)	**0.8690**	0.6433	0.3244
*D*_*ω*_(*Q*_3_, *R*_*j*_)	0.5690	**0.8779**	0.7140
*D*_*ω*_(*Q*_4_, *R*_*j*_)	0.3353	0.8621	**0.9402**
*D*_*ω*_(*Q*_5_, *R*_*j*_)	0.6516	0.8497	**0.9742**
*D*_*ω*_(*Q*_6_, *R*_*j*_)	0.4813	**0.9487**	0.8292

From Table 7, the clinical initial evaluations of the six patients demonstrate that the patient *Q*_2_ has mild BPH symptom, the patients *Q*_1_, *Q*_4_, and *Q*_5_ have severe BPH symptoms, and the patient *Q*_3_ and *Q*_6_ have moderate BPH symptoms since the patients regarding the largest measure values indicates their fit evaluation results.

To show the effectiveness of the proposed new evaluation method for the six BPH patients, we have to compare the proposed new evaluation method with the existing common evaluation method based on I-PSS^19,20^. In this case, if we do not consider the hesitant values in Table 5 for convenient comparison with the common evaluation method, the BPH symptom response values of the six BPH patients in Table 5 are reduced to the uncertain values in Table 8. Thus, the common evaluation method in the current clinical application can be applied to the BPH symptom evaluation problems of the six BPH patients in the clinical actual cases. Based on Tables 1 and 2, the totally scoring values regarding the six BPH patients are also shown in Table 8.

**Table 8:** BPH symptom responses and totally scoring values of the six BPH patients in 5 times over the past month.

*A*_*i*_	*Q*_1_	*Q*_2_	*Q*_3_	*Q*_4_	*Q*_5_	*Q*_6_
	Answer	Answer	Answer	Answer	Answer	Answer
*A*_1_	2–4	0–1	1–3	2–4	3–4	2–3
*A*_2_	3–5	0–1	0–1	2–4	3–4	2–3
*A*_3_	2–3	1	0–2	1–3	3–5	1–2
*A*_4_	2–4	0–1	1–2	3	3–5	2–3
*A*_5_	3–5	0–1	2–4	3–4	3–5	1–2
*A*_6_	2–3	1	2–3	3	3–5	2–3
*A*_7_	2–4	0–1	1–4	3–4	2–3	2–3
Totally scoring values	16–28	2–7	7–19	17–25	20–31	12–19

For the convenient comparison, the evaluation/diagnosis results given based on the common evaluation method^19,20^ and the proposed new method are indicated in Table 9.

**Table 9:** The evaluation/diagnosis results given based on the common evaluation method^19,20^ and the proposed new method.

Evaluation method	*Q*_1_	*Q*_2_	*Q*_3_	*Q*_4_	*Q*_5_	*Q*_6_
Common evaluation method^19,20^	Moderate/severe	Mild	Mild/moderate	Moderate/Severe	Severe	Moderate
The proposed new method	Severe	Mild	Moderate	Severe	Severe	Moderate

In Table 9, the common initial evaluation/diagnosis results of the six BPH patients contain or equal the ones of the proposed new evaluation method. However, the former cannot clearly indicate the diagnosis results of the three BPH patients *Q*_1_, *Q*_3_ and *Q*_4_ and implies the evaluation/diagnosis indeterminacy so as to difficultly evaluate/diagnose them in this situation; while the latter can clearly indicates its evaluation results and shows its effectiveness and rationality. Therefore, the proposed new evaluation method based on the Dice measure of CHFSs can overcome the insufficiency of the existing simply scoring evaluation method with uncertain values (i.e., the common evaluation method without the hesitant information in^19,20^).

Compared with the evaluation approach using exponential similarity measure of SNSs presented in^10^, the proposed evaluation method using the Dice measure of CHFSs contains uncertain and hesitant assessment information of patients, which the evaluation approach in^10^ cannot carry out. Furthermore, the new evaluation method in this study is very fit for patients’ thinking and expressing habits in their clinical inquiries and answers, which show the main advantage. In the BPH evaluation process, it is obvious that the proposed new evaluation method is more feasible and effective and superior to the existing initial evaluation methods^10,19,20^.

## Conclusions

Regarding the hybrid form of both interval-valued fuzzy information and hesitant fuzzy information, a CHFS is very fit for the expression of the hybrid information. Hence, this study firstly proposed the Dice measure of CHFSs based on the LCMC extension method for HFSs in CHFSs. Next, an initial evaluation approach of BPH symptoms was developed by using the Dice measure of CHFSs in CHFS setting. Lastly, the initial evaluations of six BPH patients are presented as the clinical actual cases to show the effectiveness and suitability of the proposed evaluation approach in CHFS setting.

However, the existing initial evaluation approaches^10,19,20^ cannot cope with the evaluation/diagnosis problems along with CHFS information and may lose much useful information (hesitant fuzzy information) in the evaluation process so as to result in uncertain or difficult assessment results. By comparison with existing assessment approaches, the main advantages of this study indicate (1) CHFS is very fit for the expression of uncertain and hesitant fuzzy responses of patients in the clinical assessment process; (2) The Dice measure of CHFSs based on the LCMC extension method shows the objective extension operation without the subjective extension form depending on decision makers’ preference; and (3) the developed initial evaluation approach of BPH symptoms can effectively cope with medical diagnosis problems along with uncertain and hesitant fuzzy information.

In the future, this study will be extended to other medical evaluation/diagnosis problems, such as kidney cancer and gastric cancer, in CHFS setting.

## References

[1] Adlassnig KP (1986). Fuzzy set theory in medical diagnosis. IEEE Transactions on Systems, Man, and Cybernetics.

[2] Khanale PB, Ambilwade RP (2011). A fuzzy inference system for diagnosis of hypothyroidism. Journal of Artificial Intelligence.

[3] Choi H, Han K, Choi K, Ahn J (2016). A fuzzy medical diagnosis based on quintiles of diagnostic measures. Journal of Intelligent & Fuzzy Systems.

[4] Choi G, Mun J, Ahn C (2012). A medical diagnosis based on interval valued fuzzy set. Biomedical Engineering: Applications, Basis and Communications.

[5] De SK, Biswas R, Roy AR (2001). An application of intuitionistic fuzzy sets in medical diagnosis. Fuzzy Sets and Systems.

[6] Own CM (2009). Switching between type-2 fuzzy sets and intuitionistic fuzzy sets: An application in medical diagnosis. Applied Intelligence.

[7] Ahn JY, Han KS, Oh SY, Lee CD (2011). An application of interval-valued intuitionistic fuzzy sets for medical diagnosis of headache. International Journal of Innovative Computing, Information and Control.

[8] Ye J (2015). Improved cosine similarity measures of simplified neutrosophic sets for medical diagnoses. Artificial Intelligence in Medicine.

[9] Ye J, Fu J (2016). Multi-period medical diagnosis method using a single valued neutrosophic similarity measure based on tangent function. Computer Methods and Programs in Biomedicine.

[10] Fu J, Ye J (2017). Simplified neutrosophic exponential similarity measures for the initial evaluation/diagnosis of benign prostatic hyperplasia symptoms. Symmetry.

[11] Ye S, Ye J (2014). Dice similarity measure between single valued neutrosophic multisets and its application in medical diagnosis. Neutrosophic Sets and Systems.

[12] Ye S, Fu J, Ye J (2015). Medical diagnosis using distance-based similarity measures of single valued neutrosophic multisets. Neutrosophic Sets and Systems.

[13] Broumi S, Deli I (2014). Correlation measure for neutrosophic refined sets and its application in medical diagnosis. Palestine Journal of Mathematics.

[14] Broumi S, Smarandache S (2015). Extended Hausdorff distance and similarity measure of refined neutrosophic sets and their application in medical diagnosis. Journal of New Theory.

[15] Muthumeenakshi M (2016). An application of pentagonal valued hesitant fuzzy set in medical diagnosis. Research J. Pharm. and Tech..

[16] Farhadinia B (2016). Utility of correlation measures for weighted hesitant fuzzy sets in medical diagnosis problems. Mathematical Modelling and Applications.

[17] Jun YB, Kim CS, Yang KO (2012). Cubic sets. Ann. Fuzzy Math. Inform..

[18] Fu J, Ye J, Cui WH (2018). An evaluation method of risk grades for prostate cancer using similarity measure of cubic hesitant fuzzy sets. Journal of Biomedical Informatics.

[19] AUA Practice Guidelines Committee. American Urological Association Guideline on the Management of Benign Prostatic Hyperplasia American Urological Association Education and Research, Inc. (2003).

[20] AUA Practice Guidelines Committee. American Urological Association Guideline on the Management of Benign Prostatic Hyperplasia (BPH) (Revised version). American Urological Association Education and Research, Inc. (2010).

[21] Torra, V. & Narukawa, Y. On hesitant fuzzy sets and decision. In Proceedings of the 18th IEEE International Conference on Fuzzy Systems, Jeju Island, Korea, pp. 1378–1382, 20–24 August 2009.

[22] Torra V (2010). Hesitant fuzzy sets. Int. J. Intell. Syst..

